# Chinese Herbal Therapy for Chronic Tension-Type Headache

**DOI:** 10.1155/2015/208492

**Published:** 2015-06-14

**Authors:** YanQing Tong, LiXiang Yu, Ye Sun

**Affiliations:** ^1^Department of Neurology, The First Affiliated Hospital to Changchun University of Chinese Medicine, Gongnong Road, No. 1478, Changchun, Jilin 130021, China; ^2^The First Affiliated Hospital to Changchun University of Chinese Medicine, Changchun 130021, China

## Abstract

*Objective*. To investigate the effects of Chinese herbal therapy on chronic tension-type headache. *Method*. 132 patients with chronic tension-type headache were enrolled in the study. All patients filled in headache questionnaire at baseline phase and 4, 8, and 12 weeks after baseline. As an alternative therapeutic method, the patients were orally administrated Chinese herbal concoction for ten days. Therapeutic effects were evaluated during 12 weeks of followup. *Result*. In the primary outcome analysis, mean headache scores were significantly lower in the group. Scores fell by 25%–40% during 12 weeks of followup. Patients fared significantly well for most secondary outcome measures. From baseline to 4–12 weeks of followup, the number of days with headache decreased by 6.8–9.5 days. Duration of each attack also significantly (*P* < 0.05) shortened from 5.3 hours at 4 weeks to 4.9 hours after 8 weeks of followup. Days with medication per four weeks at followup were lower than those at the baseline. The differences were significant (*P* < 0.05, 0.01) for all end points. Days with medication fell by 56.6% at 12 weeks. *Conclusion*. The study has provided evidence that Chinese herbal therapy can be clinically useful for the treatment of chronic tension-type headache.

## 1. Introduction

Every year the lives of many people throughout the world are affected by headaches. Tension-type headache is classified as episodic if it occurs on less than 15 days a month and as chronic if it occurs more often [1]. Episodic tension-type headache can be treated with rest and analgesics, while chronic tension-type headache demands a more fundamental treatment [2]. Chronic tension headache represents a considerable social burden in terms of both costs to the health services and also the costs of lost productivity [3–5]. Despite the undoubted benefits of medication, many chronic tension-type patients continue to experience distress and social disruption. This leads to alternative approaches to headache care. One of the approaches seems to be Chinese herbal therapy.

While some researchers recommended that Chinese herb is valuable for various types of headache, including chronic tension-type [6–8], the data was limited. In this study, Chinese herbs were used with the aim of exploring their effect on chronic tension-type headache.

## 2. Methods

The study was a clinical trial performed at outpatient department in the First Affiliated Hospital to Changchun University of Chinese Medicine, China, from 3rd of March, 2011, to 18th of December, 2012. The study protocol was approved by the Research Ethics Committee of the Hospital (Approval number CC201102).

Patients were selected consecutively by the neurologists of the outpatient department, according to the inclusion and exclusion criteria below. Protocol summaries were reviewed by the participants, and written informed consents were obtained on the day of the study after a detailed explanation of the study purpose and methods. Those who were eligible and willing to participate were assessed by an independent physician. This assessment included a detailed history, physical examination, and collection of baseline data. All patients filled in headache questionnaire at baseline phase and 4, 8, and 12 weeks after baseline. As the main outcome measures, the headache questionnaire included analogue scale of headache score on a scale from zero (no pain) to 10 (most severe pain), duration of each attack (in hours), and the number of days on which headaches occurred per four weeks.

The main inclusion criterion was chronic tension-type headache diagnosed by criteria of International Headache Society [9], for which the subject had not received any treatment in the previous one week, besides symptomatic medication. Patients were excluded for any of the following: onset of headache disorder less than one year before; patients who had papilloedema, or pulsating headaches, or asymmetrical pupillary reflexes, or neurological deficits, or systemic disorders; pregnancy; and patients with creatinine, serum glutamic oxaloacetic transaminase (SGOT), or alkaline phosphatase levels 50% greater than the upper limit of normal for the investigator's laboratory.

At the first visit, all patients underwent initial assessment and completed questionnaires. Following this, as an alternative therapeutic method, the patients were orally administrated Chinese herbal concoction. The prescription was as follows: Tu Fuling (*Smilax glabra* Roxb) 30 g, Jin Yinghua (*Lonicera japonica* Thunb) 20 g, Deng Xincao (*Juncus effusus* L. var. decipiens Buchen) 15 g, Yuan Husuo (*Corydalis yanhusuo* W.T. Wang) 15 g, Man Jingzi (*Vitex trifolia* L.var.simplicifolia Cham.) 15 g, Fang Feng (*Saposhnikovia divaricata* (Turcz.) Schischk) 15 g, Tian Ma (*Gastrodia elata* Bl.) 15 g, Chuan Xiong (*Ligusticum wallichii* Franch.) 20 g, Bai Zhi (*Angelica dahurica* (Fisch. ex Hoffm.) Benth. et Hook. J. ex Franch.ex Sav.) 15 g, and Xin Yi (*Magnolia liliflora* Desr.) 3 g. The concoction was prepared by mixing the crude drugs in 800 mL water, getting 200 mL liquor after the drugs are decocted in 800 mL water (100°C for 30 minutes twice). After cooling, concoction was stored with temperature 18–24°C, humidity 55%–70%. The concoction was orally administrated by 200 mL/day, 100 mL twice per day, for ten days.

Laboratory tests that had been performed at baseline (complete blood cell count; SGOT, serum glutamic pyruvic transaminase (SGPT), alkaline phosphatase, and serum creatinine determinations) were repeated after the treatment. Subjective data were collected from daily diaries that were given to patients at each treatment session and collated by one observer.

Therapeutic effects were evaluated during 12 weeks of followup. Assessments were made at baseline and every 4 weeks up to 12 weeks. The primary outcome measures were the headache score at 12-week followup. Secondary outcome measures included the duration of each attack (in hours), the days with headache in 4 weeks [10], and use of medication scored with the medication quantification scale [11].

### 2.1. Statistical Analysis

Statistical methods used included paired *t*-tests for comparison of mean values. All analyses were carried out using SPSS Statistics 19.0. *P* < 0.05 was considered statistically significant.

## 3. Results

**Figure 1:**
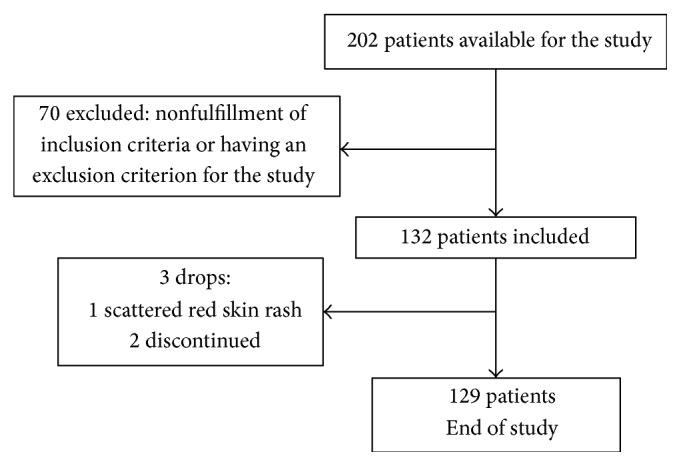
Flow chart of entry and discontinuation by patients during the study.

A total of 132 patients with chronic tension-type headache, aged 26–55, who met the inclusion criteria, were included (Figure 1). Three patients did not complete therapy. One patient developed scattered red skin rash 3 days after the first oral administration. This patient discontinued the therapy. Two days after the discontinuation, the skin rash disappeared. The rate of adverse events was 0.76% in the group. The other two participants discontinued for no therapy-related reasons. No clinically significant changes in hematologic or biochemical laboratory parameters were identified in laboratory monitoring.

The baseline characteristics are shown in Table 1.  Table 2 summarizes the results for medical outcomes for patients completing 12 weeks of followup at baseline and 4, 8, and 12 weeks after baseline. There were significant changes over time after therapy. In the primary outcome analysis, mean headache scores were significantly lower in the group. Scores fell by 25%–40% during 12 weeks of followup compared with baseline. But the effects of Chinese herbs did not seem to be long lasting. Headache scores were slightly higher at 12 weeks than 4 weeks after treatment. Patients fared significantly well for most secondary outcome measures. From baseline to 4–12 weeks of followup, the number of days with headache decreased by 6.8–9.5 days in the group. Duration of each attack also significantly (*P* < 0.05) shortened from 5.3 hours at 4 weeks to 4.9 hours after 8 weeks of followup. Days with medication per four weeks at followup were lower than those at the baseline. The differences were significant (*P* < 0.05, 0.01) for all end points. Days with medication fell by 56.6% at 12 weeks.

## 4. Discussion

This trial assesses the key variables of headache in patients with chronic tension-type headache given herbal therapy. Herbal therapy results in clinically relevant benefits for patients with chronic headache. We also found decreases in use of medication. There was a significant improvement compared to the baseline for each time point. Symptoms of chronic tension-type headache abated significantly, and the effects were sustained through the followup period of 12 weeks. Methodological strengths of our study include a large sample size and high follow-up rates.

Safety is an important consideration in the management of chronic conditions such as headache. The common pharmacological therapies, such as metoprolol and flunarizine, have associated side effects, including drowsiness, ataxia, and blushing [12, 13]. In this analysis, we found a low incidence of side effects, which were related to mild allergic reaction.

There were some limitations. For lack of similar herbal drugs, our study did not have control group. One hypothesis might be that the effects seen resulted not from the action of herbal therapy but from the “placebo effect.” Nonetheless, the effects of Chinese herbs were sustained during the 12 weeks of followup. This implies that our findings perhaps cannot be explained purely in terms of the placebo effect.

Patients recorded the use of analgesics for headache during the course of the study, which were lower after therapy, indicating that the superior results were not due to influence of effective cointerventions.

Chinese herbal medicine is a method of treatment rooted in an ancient Chinese culture that has existed for at least two millennia. Although the exact underlying neurophysiological mechanisms remain unclear, the results suggest that herbal therapy provides neuromodulating effects.

Traditional Chinese Medicine (TCM) is a system of healing that originated thousands of years ago. It has evolved into a well-developed, coherent system of medicine that uses several modalities to treat and prevent illness. The philosophy behind TCM revolves around the balance of the Yin and Yang. The Yang energy tends to flow up. In TCM, a common internal cause of headache is (1) liver yang rising up to the head, as a result of long-term deficiency of liver yin; (2) liver fire, a condition of extreme heat, liver fire going in an upward direction in the body leading to excess in the upper part of the body; and (3) qi stagnation and blood stasis. The Yang meridians intersect in the head. The above incentives lead to a blockage of the Yang meridians and cause headaches. When qi stagnation and blood stasis start to set in, the headache gets worse [14].

Chinese herbs applied are to lead the liver yang to flow downward, regulate the qi, and disperse blood stasis. Herbal therapy works to clear the blockages of the Yang meridians, harmonize the organs, and reestablish a balance of Yin and Yang.

The current study has provided evidence that Chinese herbal therapy can be clinically useful for the treatment of chronic tension-type headache.

## Figures and Tables

**Table 1:** Baseline characteristics.

Characteristics	
All patients (*n*)	132
Male [*n* (%)]	35 (26.5)
Age [mean (SD)]	40.5 (13.2)
Body mass index [mean (SD)]	23.3 (3.5)
Duration (years) [mean (SD)]	8.5 (2.8)
Days with headache per four weeks [mean (SD)]	16.0 (0.5)
Duration of each attack (hours) [mean (SD)]	9.8 (3.5)
Headache score [mean (SD)]	6.0 (3.3)
Days with medication per four weeks [mean (SD)]	13.6 (2.2)

**Table 2:** Outcome measures.

Outcome measures	Baseline	4 weeks	8 weeks	12 weeks
Headache score	6.0 (3.3)	3.6 (2.1)^*∗*^	3.8 (3.0)^*∗*^	4.5 (3.5)
Duration of each attack (hours)	9.8 (3.5)	4.5 (3.0)^*∗*^	4.9 (3.8)^*∗*^	5.6 (4.1)
Days with headache per four weeks [mean (SD)]	16.0 (5.2)	6.5 (6.0)^#^	7.8 (4.3)^*∗*^	9.2 (5.5)
Days with medication per four weeks [mean (SD)]	13.6 (2.2)	4.5 (3.9)^#^	4.8 (2.8)^#^	5.9 (2.3)^*∗*^

Paired *t*-tests: ^*∗*^ *P* < 0.05; ^#^ *P* < 0.01.
